# Hepatic small vessel neoplasm, a first case report in a liver transplant recipient

**DOI:** 10.3389/fgstr.2026.1956520

**Published:** 2026-10-26

**Authors:** Afaf Awad Y. Ahmad, Michael Craughwell, Lindsey Clarke, Niamh Nolan, Audrey Dillon, Zita Galvin

**Affiliations:** 1 The National Liver Transplant Unit, St. Vincent’s University Hospital, Dublin, Ireland; 2 Histopathology Department, St. Vincent’s University Hospital, Dublin, Ireland

**Keywords:** CMV, CT, EBV, H&E, HSVN

## Abstract

Hepatic small vessel neoplasm (HSVN) is a recently recognized vascular tumor of the liver that behaves in a largely indolent fashion but carries uncertain malignant potential. Unlike hepatic angiosarcoma, HSVN lacks marked cytologic atypia and does not demonstrate diffuse p53, GLUT1, or c-Myc overexpression. We describe what appears to be the first reported case of HSVN arising in a liver allograft, highlighting diagnostic challenges and management considerations in a transplant recipient.

## Background

Hepatic small vessel neoplasm (HSVN) is an uncommon hepatic vascular tumor that has only been characterized in the past decade. Although its infiltrative pattern may resemble angiosarcoma, HSVN demonstrates a distinctly benign cytologic profile and consistently negative staining for markers typically associated with aggressive vascular malignancies. Because of its rarity and overlapping radiologic features with other hepatic lesions, diagnosis relies heavily on histopathology.

## Case presentation

A 65-year-old man underwent orthotopic liver transplantation for hepatic failure due to systemic amyloid light-chain (AL) amyloidosis (kappa light chain). His medical history was notable for renal amyloidosis requiring hemodialysis and subsequent renal transplantation in the second year post liver transplant, secondary polycythemia, atrial fibrillation, hypertension, factor X deficiency, and non-amyloid congestive cardiac failure. He remained on long-term immunosuppression and hematologic therapy including tacrolimus, mycophenolate mofetil, prednisolone, and daratumumab.

He was followed regularly in a multidisciplinary transplant clinic and maintained excellent graft function.

In the sixth year post liver transplant, he reported nonspecific abdominal bloating and discomfort without abnormal findings on examination.

He had been evaluated in the same year for mildly elevated liver enzymes with imaging and biopsy, which showed no recurrent amyloid and no focal hepatic lesions.

## Investigations

Routine laboratory tests remained stable, including normal renal function for his graft, preserved synthetic liver function, and alpha-fetoprotein (AFP) <2.3. Viral screening for cytomegalovirus (CMV), Epstein–Barr virus (EBV), HIV, and hepatitis B virus (HBV) were negative.

A repeat CT of the thorax, abdomen, and pelvis was performed for persistent abdominal symptoms and revealed a new 1.2-cm arterially enhancing lesion in segment VI of the transplanted liver (**Figure 1**). This lesion had not been present on imaging 3 years earlier.

**Figure 1:**
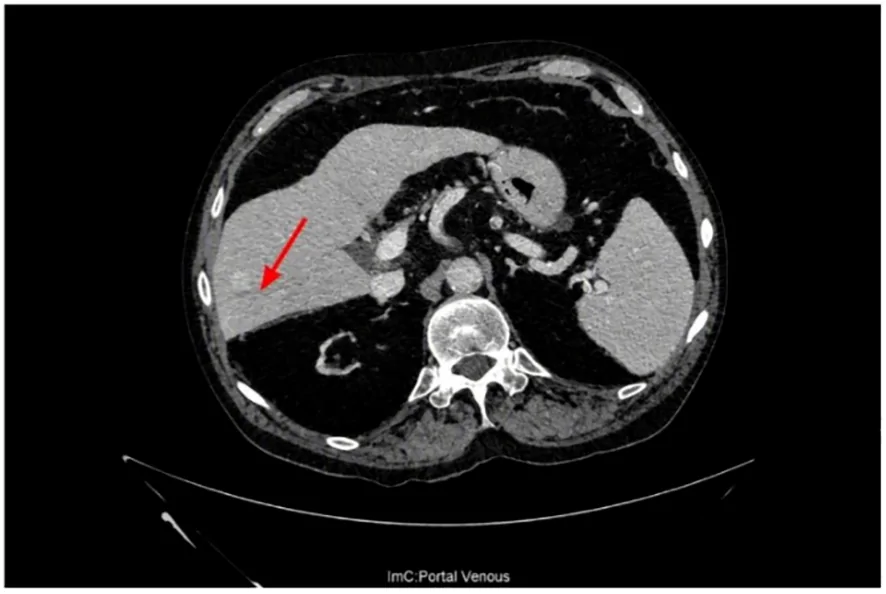
Contrast-enhanced CT shows a new 1.2-cm lesion with arterial enhancement in segment VI of the liver.

The findings were discussed by the radiology multidisciplinary team (MDT), which noted a new 1.2-cm arterially enhancing lesion that had not been present on the CT performed 3 years earlier. Given his gastrointestinal symptoms, the team considered bidirectional endoscopy was appropriate. The lesion was also considered unusual and a possible metastasis from another site was considered. The MDT also recommended going straight to a liver biopsy, which we organized.

Endoscopic evaluation (gastroscopy and colonoscopy) was unremarkable, with biopsies showing normal mucosa and no amyloid.

## Histopathology

Ultrasound-guided biopsy of the lesion demonstrated the following (**Figure 2**): Fragmented cores of liver with two portal tracts without significant inflammation. A short core of tissue with back-to-back vessels was present and separate from the other liver fragments. There was no necrosis; the lining endothelial cells were plump but not pleomorphic; and no mitoses were identified. They were positive for CD31 and for CD34. P53 was wild type. Ki67 index was 8%. No intra-lesional hepatocytes were identified. The background liver did not include portal tracts and was too small for architectural assessment. No inflammation was seen and there was no evidence of amyloid deposition. In conclusion, the appearances were of a hepatic small vessel neoplasm.

**Figure 2:**
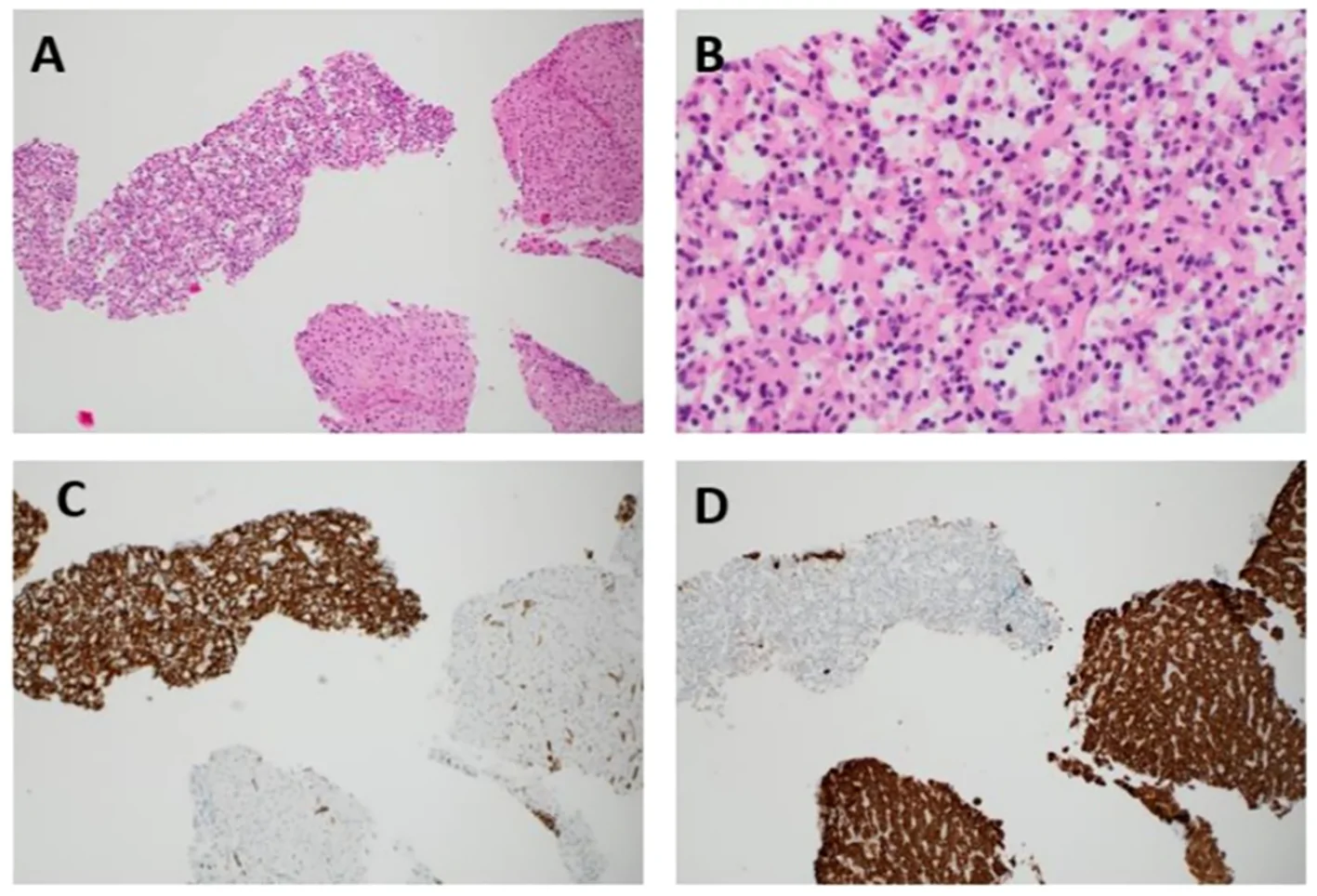
Histopathology. **(A)** H&E ×10, showing background liver. **(B)** H&E ×40, showing the neoplasm. **(C)** CD34 ×10, highlighting the neoplasm. **(D)** HepPar-1 ×10, highlighting the background liver, with no staining in the neoplasm.

This was generally regarded as a vascular neoplasm of unknown malignant potential based on the histopathologist’s report. The MDT had suggested follow-up with an ultrasound in 6 months’ time, provided the lesion is visible on ultrasound.

## Multidisciplinary discussion

The lesion was reviewed at the hepatobiliary histopathology and radiology MDTs. Given its small size, benign histologic appearance, and the patient’s complex comorbidities, the consensus was to monitor the lesion with interval imaging. Ablation, transarterial chemoembolization (TACE), or surgical resection were reserved for potential future growth.

Follow-up ultrasound at 3 months demonstrated a stable 1.2-cm lesion with no new abnormalities. Serial imaging over subsequent months continued to show stability, and the patient remained asymptomatic.

## Discussion

Over the last decade, hepatic small vessel neoplasms have been identified as a discrete entity among hepatic vascular neoplasms, exhibiting a benign pattern with uncertain malignant potential but lacking the aggressive, marked cytologic atypia of angiosarcomas and showing no significant p53, GLUT1, or c-Myc immunostaining.

HSVNs were first reported in 2012–2013 as case reports. Over the following 3 years, more cases were collected and officially described by Gill et al. In 2016, cases identified at the University of California, San Francisco, were described as a distinct entity characterized by small vessels and infiltrative borders (1).

They were initially defined as hepatic vascular tumors of uncertain malignant potential that may be mistaken for angiosarcomas owing to an infiltrative growth pattern but lack the marked cytologic atypia of some angiosarcoma cases. However, they did not show significant p53, GLUT1, or c-Myc immunostaining, all of which were diffusely positive in a subset of hepatic angiosarcomas (1, 2).

It is characterized by strong expression of vascular markers (CD31, CD34, FLI-1, and ERG) and is pancytokeratin negative. All cases have a low Ki-67 proliferation index (<10%) (3).

In the vast majority of cases, HSVN presented as a single lesion in the liver. However, in four patients included in a review by Paisant et al., there was evidence of a second hepatic lesion, thus suggesting that HSVN may occur as a multifocal disease (4).

It is still challenging to characterize HSVN radiologically because no clear defining radiological criteria are known. In our case, arterial enhancement was described. Similarly, Paisant et al. reported a case series involving four patients who underwent contrast-enhanced ultrasound (CEUS), computed tomography, and MRI. All modalities demonstrated similar enhancement patterns on arterial phase imaging with early, irregularly thick peripheral and continuous enhancement. The appearance at the arterial phase on larger lesions was described as “flower petal-shaped” due to early septal enhancement. Imaging-specific characteristics include persistent thick rim enhancement with mild central washout on CEUS and heterogeneous hyperintensity on T2-weighted imaging (4). However, there has been a case with innumerable enhancing lesions that were only visualized on the arterial phase (5).

Imaging of the other documented HSVN cases was also inconclusive, and impressions ranged from atypical vascular tumor to neuroendocrine tumor to hepatocellular carcinoma (HCC); biopsy was clearly needed for diagnosis (1, 2). One differential diagnosis is hemangioma, as both HSVN and hemangioma might show similar homogeneous acoustic enhancement on ultrasound; however, on contrast-enhanced CT, HSVN lacks the typical central enhancement in the delayed phase, with persistent homogeneous enhancement as shown in **Figure 1**.

Most cases in the literature were idiopathic, with no specific toxin or drug exposure identified on review of HSVN patients’ histories (1, 2).

Unique to our case was the discovery of this HSVN in a liver transplant recipient, a setting not previously described in the literature. Predisposing factors and the behavior of these neoplasms in such a cohort, along with the immunosuppressive effects, will require further investigation and research, as increasing numbers of transplants and advances in diagnostics will likely reveal more cases.

Currently, there are no clear management guidelines available. In our case, in view of the age of the patient, history of liver and renal transplantation, and significant burden of his comorbidities, a monitoring strategy was initiated following multidisciplinary discussions, with ablation, TACE, or resection considered only if there is evidence of growth. However, to date, follow-up imaging has confirmed a stable liver lesion, and the patient remains under imaging surveillance every 6 months.

All the cases in the literature were managed case by case, and the majority of cases underwent resection (1, 2, 6). In terms of prognosis, it is noted that HSVN has a low recurrence rate regardless of margin status. In the original description by Gill et al, follow-up data were available in 12 patients (range: 1–24 months). There was no evidence of disease progression in any of the patients, despite incomplete excision in some cases (1, 2, 6). In contrast, Di Salvatore et al. described a multifocal HSVN with splenic dissemination that was managed conservatively; their patient had an uneventful 15-month follow-up, and the hepatic and splenic lesions were stable in number and size. They concluded that, at the time of the report, there was no evidence of recurrence or progressive disease in any of the patients for whom follow-up was available. Longer follow-up is needed to establish the potential for recurrence or metastasis of this entity (7, 8).

## Data Availability

The original contributions presented in the study are included in the article/supplementary material. Further inquiries can be directed to the corresponding author.

## References

[1] Gill RM Alves V Makhlouf H Sempoux C Thung S Ferrell LD . Small vessel hepatic hemangioma, an atypical hemangioma variant in adult liver. Mod Pathol. (2012) 25:413A.

[2] Gill RM Sempoux C Makhlouf H Thung S Amancio TT Alves V . GLUT1 expression in adult hepatic vascular neoplasms. Mod Pathol. (2013) 26:402A.

[3] Cicala CM Monaca F Giustiniani MC Di Salvatore M . Multifocal hepatic small vessel neoplasm with spleen dissemination. BMJ Case Rep. (2022) 15:e248785. doi: 10.1136/bcr-2022-248785 35351763 PMC8966554

[4] Paisant A Bellal S Lebigot J Canivet CM Michalak S Aube C . Imaging features of hepatic small vessel neoplasm: case series. Hepatology. (2021) 74:2894–6. doi: 10.1002/hep.31779 34131938

[5] Akanuma N Joseph NM Stachler M Behr S Takahashi T Gill RM . Malignant hepatic vascular neoplasm with novel RAF1 and GNA11 mutations: risk stratification considerations for hepatic small vessel neoplasm (HSVN). Hum Pathol Rep. (2022) 29:300671. doi: 10.1016/j.hpr.2022.30067142574925

[6] Goh IY Mulholland P Skolova A liu C Siriwardhane M . hepatic small vessel neoplasm- A systematic review. (2021). doi: 10.1026/j.amsu.2021.103004 PMC8591473 34815856

[7] Cicala CM Monaca F Guistiniani MC Di Salvatore M . Multifocal hepatic small vessel neoplasm with spleen dissemination a case report. In: BMJ Case Reports (2022). doi: 10.1136/bcr-2022-248785 PMC8966554 35351763

[8] Miranda M Howell D El Jabbour T . Hepatic small vessel neoplasm: not totally benign, not yet malignant. (2023). doi: 10.4132/jptm.2023.06.19 PMC10518243 37608553

